# A Stab in the Dark: A Case Report of an Atypical Presentation of Giant Cell Arteritis (GCA)

**DOI:** 10.3390/geriatrics3030036

**Published:** 2018-06-29

**Authors:** Beth McCausland, David Desai, David Havard, Yasmin Kaur, Asalet Yener, Emma Bradley, Harnish P. Patel

**Affiliations:** 1 Medicine for Older People, University Hospital Southampton, Southampton SO16 6YD, UK; Beth.McCausland@uhs.nhs.uk (B.M.); David.Desai@uhs.nhs.uk (D.D.); dwhavard@hotmail.co.uk (D.H.); yasminkaur@nhs.net (Y.K.); Asalet.Yener@uhs.nhs.uk (A.Y.); Emma.Bradley@uhs.nhs.uk (E.B.); 2 Academic Geriatric Medicine, University of Southampton, University Hospital, Southampton SO16 6YD, UK; 3 NIHR Biomedical Research Centre Southampton, University of Southampton, Southampton SO16 6YD, UK

**Keywords:** non-specific symptoms, inflammation, sepsis, giant cell arteritis, temporal arteritis, multidisciplinary team working, temporal artery biopsy, FDG-PET/CT

## Abstract

**Background:** Giant cell arteritis/temporal arteritis (GCA) is an inflammatory condition that affects large to medium vessels such as the aorta and its primary branches. Patients classically present with fatigue, fever, headache, jaw claudication and in severe cases, may suffer either transient (amaurosis fugax) or permanent visual loss. The reference standard for diagnosis is the temporal artery biopsy (TAB) and the mainstay of treatment is with immunosuppression. Our patient JG, presented with a range of non-specific symptoms that mimicked generalised sepsis, but was ultimately diagnosed with GCA through effective, methodical multi-disciplinary team (MDT) work. **Clinical case:** JG, an 81 year old gentleman, presented acutely with a 3–4 weeks history of fatigue, lethargy, pyrexia and a marked inflammatory response suggestive of a sepsis but without a clear primary source or clinical features of vasculitis. His inflammatory markers were markedly raised although his erythrocyte sedimentation rate (ESR) was not elevated. He was initially treated for sepsis of unknown origin however, body imaging after admission suggested a possible infection around a previous aortic graft site. This was refuted in subsequent 18F-fluorodeoxyglucose-positron emission tomography (FDG-PET/CT) scanning. Microbiological, parasitic, as well as autoimmune assays were unremarkable. He underwent a TAB which was diagnostic for GCA and as a result, was started on oral corticosteroids with immediate symptom relief. He was discharged and followed up on an outpatient basis. **Conclusions:** This case highlights how a vasculitis can present with a range of non-specific symptoms that may resemble a fever of unknown origin (FUO)/sepsis that can lead to a delay in making the correct diagnosis. It also highlights the importance of considering a diagnosis of vasculitis in patients who present with a FUO where there is no clear focus of infection. Delays in diagnosis and management of these conditions can potentially lead to significant irreversible morbidity.

## 1. Introduction

This unique case highlights that patients presenting with non-specific symptoms in whom inflammatory markers suggest a generalised sepsis/FUO, but have no clear microbiological source, a differential diagnosis of vasculitis should be entertained. Appropriate non-invasive and invasive investigations as well as medical management strategies should be instituted immediately in view of the morbidity i.e., permanent loss of vision, associated with this condition. In our patient, multi-disciplinary team (MDT) working and involvement of specialty colleagues e.g., rheumatology and microbiology/infectious diseases teams was invaluable. This case report has been written in conjunction with the CARE (CAse REport) checklist [1].

## 2. Case Presentation

JG, an 81 year old, non-smoking, independently active, Caucasian male presented with a vague, two to three week history of generalised malaise, myalgia and decreased physical function which were preceded by coryzal symptoms associated with episodic pyrexia, a sore throat and cough. His past medical history included a graft repair of an abdominal aortic aneurysm 13 years prior to his current admission and hypertension. His regular medication on admission included Losartan 100 mg once daily and Pravastatin 10 mg once daily. There was no history of recent travel although his previous occupation had included staying for long spells in Asia and Africa several decades previously. There was no history of visual disturbances, headaches, jaw ache, nasal congestion or history of skin rashes on systems examination.

On admission, he had a haemoglobin of 138 g/L, a raised white blood count of 26.2 × 10^9^/L that was predominantly neutrophilic with toxic degranulation on blood film, an erythrocyte sedimentation rate (ESR) of 22 mm/h, eosinophilia at 1.7 × 10^9^/L, and a c-reactive protein (CRP) of 245 mg/L. His urea and creatinine were elevated at 24.2 mmol/L and 154 μmol/L from a baseline of 8.2 mmol/L and 126 μmol/L, respectively. His alkaline phosphatase (ALP) was 185 U/L, from a baseline of 82. His creatine kinase (CK) was 83 U/L and he had a low albumin of 19 g/L (Table 1).

A sepsis of unknown origin was suspected however, there were no significant findings on a screen that comprised a urine analysis, chest radiography, and serial blood cultures.

He was initially managed with empirical broad spectrum antibiotics and intravenous fluids for the kidney injury. After one week with little clinical improvement and several episodes of fever, a computerised tomography (CT) scan of the abdomen and pelvis was performed to determine a source for his systemic inflammatory response. This revealed ill-defined soft tissue surrounding the abdominal aorta at the level of the aortic repair suspicious for an inflammatory process/infection of the aortic graft. Simultaneously, and especially due to his marked inflammatory response that included a neutrophilia and eosinophilia that had peaked at a level of 6.5 × 10^9^/L, a transthoracic echocardiogram, an autoantibody screen including anti-neutrophil cytoplasm antibody (ANCA) and investigations for haemolytic anaemia were requested and were subsequently unremarkable. Similarly, tests for HIV, Lyme disease, Syphilis, Epstein Barr virus (EBV), cytomegalovirus (CMV), Hepatitis, Legionella, Mycoplasma, Strongyloides, and thyroid dysfunction were all negative.

**Figure 1:**
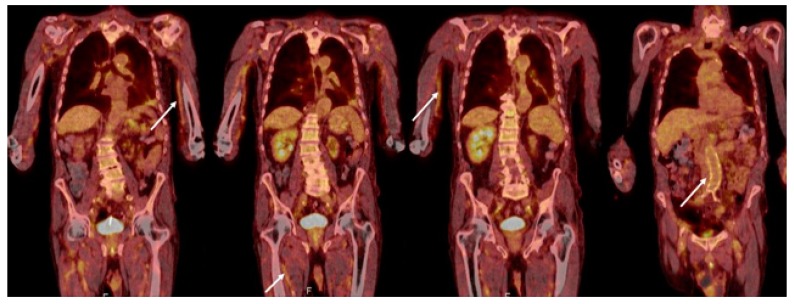
An 18F-fluorodeoxyglucose-positron emission (FDG-PET/CT) scan. Appearances were nonspecific with low grade increased tracer uptake in the arterial tree most prominent in both upper limbs and circumferentially around a non-dilated abdominal aorta, pelvis, and upper thighs (arrows). The causes of these appearances included a ‘blood pool effect’ related to timing of scan or diffuse low-grade vasculitis. Some focal activity in the partly consolidated left lower lobe of lung and related small left pleural effusion were reported as potential sites of infection.

**Figure 2:**
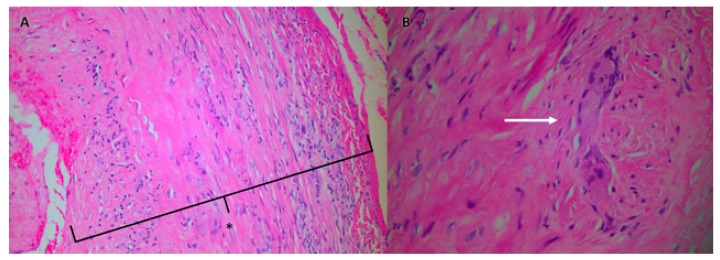
Histology specimen obtained from the patient’s temporal artery biopsy that was typical of temporal (giant cell) arteritis. Panel **A** shows an inflammatory cell infiltrate with a cuff of lymphocytes and monocytes within the adventitia, inflammation across the media with involvement of an oedematous intima (*), and presence of occasional multinucleated giant cells (Panel **B**, arrow).

**Figure 3:**
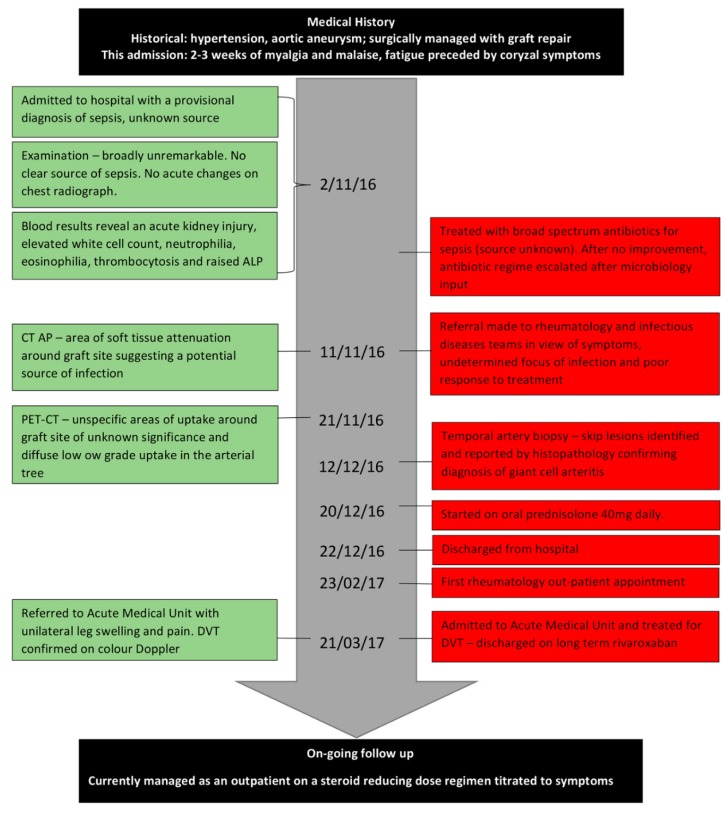
Time-line of patient JG’s journey from admission to follow up.

An 18F-fluorodeoxyglucose-positron emission tomography (FDG-PET/CT) was requested in view of the findings at the aortic graft site. It was not intended as an *a priori* investigation for a diagnosis of vasculitis. However, it revealed non-specific low grade increased tracer uptake in the arterial tree but no clear evidence of aortic graft infection (Figure 1). Given the diagnostic uncertainties, absence of a causative organism and normal serology, a MDT decision was made to proceed to a temporal artery biopsy (TAB) (a stab in the dark) without prior colour Doppler sonography (CDS) investigation. The 18 mm × 2 mm × 2 mm specimen of the temporal artery revealed an inflammatory cell infiltrate and presence of occasional multinucleated giant cells consistent with temporal (giant cell) arteritis (GCA) (Figure 2). JG was immediately commenced on 40 mg prednisolone, once a day, along with calcium, vitamin D, and a bisphosphonate for bone protection. He was discharged home with follow up by the rheumatology team. His renal, and liver function had stabilised prior to discharge (Table 1). He adhered to the treatment with corticosteroids and was guided by the rheumatology team as to how to taper the steroid dose over time. The timeline of the patient’s investigations, management, and treatment is shown in Figure 3.

## 3. Review

Vasculitis, defined as inflammation of blood vessel walls is categorised by the size (large, medium, and small) of blood vessel affected as well as the presence or absence of anti-neutrophil cytoplasm antibodies (ANCA) [2]. The inflammatory response and consequent sequelae can cause damage or occlusion of the vessel resulting in downstream end-organ ischaemia and tissue damage. Symptoms are therefore dependent on the anatomical distribution of the vessel or vessels involved.

Large vessel vasculitis affects the large arteries and there are two major variants, Takayasu’s arteritis and Giant Cell Arteritis (GCA). Giant cell arteritis is the most common vasculitis in those aged over 50 years with a reported incidence of 20 per 100,000 people and often co-exists with Polymyalgia Rheumatica (PMR) [3]. The incidence rises with age; 74–76 year olds, women and those of Northern European, Caucasian descent being most commonly affected [4]. The aetiology is not fully known but is thought to involve an interaction between genes, environment and the immune system where a genetically predisposed individual exposed to an exogenous stimulus develops an exaggerated immune inflammatory response within the blood vessels [5]. Polymorphisms of HLA-DRB, IL-17, IL-33, and X-chromosome genes have been implicated in pathogenesis of GCA and have been investigated as risk factors for GCA. However, robust associations have not been found to allow for genetic screening [4].

Postulated exogenous stimuli include cigarette smoke and infections with varicella zoster virus (VZV). Two studies have found that smoking independently associated with GCA in women (odds ratio [OR] 5.48 (2.12–14.19), *p* = 0.00006) [6] and (OR 6.324 (3.50–11.42), *p* ≤ 0.0001) [7], but not men (OR 1.16 (0.64–2.13), *p* = 0.621) [6]. Smoking may represent a cofactor, aiding rather than triggering GCA development [6] by impairing arterial endothelium and oestrogen synthesis [7]. Smoking has also been associated with more severe ischaemic symptoms [8].

Incidence of GCA has been correlated with peaks of certain infections [9]. Theoretically, pathogenic vectors that access the vessel wall media trigger an exaggerated immune response [6]. VZV has been found in 73% of GCA biopsy positive and 64% of GCA biopsy negative temporal arteries versus 22% in control samples [10]. VZV has also been found in TAB skip lesions, adjacent to GCA pathology in amounts suggestive for a role in the immunopathogenesis [11]. However, these data stem from pre-clinical trials and cannot reliably be extrapolated for clinical use at present [4].

The cellular and humoral immune systems are both implicated in the pathogenesis of GCA. Release of pro-inflammatory IL-6 stimulates production of both Th1 and Th17 cells (which produce IL-17 and have been implicated in autoimmune disorders), inhibits T-regulatory cells and potentiates the inflammatory response [5]. B-cells overproduce autoantibodies and contribute to the production of IL-6 and development of Th17 cells. B-cell synthesis is also dysregulated, with reduced production of B-cells in proportion to increasing ESR and increased IL-6 [12]. Dendritic cells become activated, giant cells form from coalesced activated macrophages and contribute to the development of granulomata. Interferon gamma (IFN-γ) is the main mediator implicated in granuloma formation [13]. Reactive oxygen species and matrix metalloproteinases are released that damage the elastic lamina. Intimal cells are stimulated to proliferate that contribute to narrowing of the vessel lumen leading to a reduction in blood flow and predisposition to downstream ischaemic symptoms [5]. Differential cytokine release may lead to altered patterns of vascular wall and systemic inflammation that can give rise to discrepancies between CRP/ESR measurements and clinical features. For example, Keser et al. [14] postulate that there may be two subgroups of GCA; the first is characterised by severe systemic inflammation associated with a predominantly IL-6/Th17/IL-17 driven pathway and the second, a less severe systemic response but with prominent neuro-ophthalmic ischaemic complications driven by IFN-γ/IL-12/Th1 mediated effects on the vascular wall. Histology in biopsy specimens demonstrates both leucocytoclasis and fibrinoid necrosis similar to what was seen JG’s histology specimens (Figure 2).

## 4. Clinical Features

Presenting features of GCA can include headache with discomfort at the side of the head associated with swelling and/or tenderness of the temporal arteries, scalp tenderness and in many cases, systemic constitutional symptoms. Jaw or tongue claudication as a consequence of ischaemia of the masseter muscle stops patients from eating or talking and is a result of narrowing of the facial artery or its branches [15]. Claudication symptoms may herald impending blindness as the cranial predilection of the disease can involve the posterior ciliary arteries supplying the retina. Visual loss may start unilaterally but can progress to affect the contralateral eye [15]. Stroke is also a small but serious complication of GCA. As these symptoms and signs are associated with high morbidity, correct and timely diagnosis of GCA is necessary.

## 5. Investigations

Clinical examination is invaluable in the early recognition and diagnosis of GCA. Immediate treatment with high dose glucocorticoid is advised if there is a high index of suspicion and a clinical diagnosis is considered likely. Delay in treatment leads to an increased risk of retinal and cerebral complications. Initial investigations should involve a full blood count, CRP and ESR as the majority of patients will have raised inflammatory markers on presentation [16]. An ESR > 50 mm/h provides sufficient evidence along with clinical symptoms to warrant further investigation and/or treatment based on the index of suspicion. However, up to 4% of patients can have a normal ESR [17]. A study by Kermani et al. demonstrated that seven out of 177 patients whose TAB was consistent with GCA had normal ESR and CRP at diagnosis. These patients had fewer constitutional symptoms alongside a significant increase in polymyalgia rheumatica symptoms [18]. Furthermore, ESR as a measure of systemic inflammation may be reduced in the setting of co-existent infections, renal insufficiency and low albumin levels, as may have been the case in our patient [19]. Autoantibodies do not currently have a role to play in the investigation of GCA.

The prevalence of GCA masquerading as fever of unknown origin (FUO) has been estimated to range from 1% to 67% [20,21]. Constitutional fever is estimated to be present in 15% of patients with GCA [22], rising to up to 40% if associated with PMR [23].

Classification of GCA can be made by following the American College of Rheumatology criteria (ACR) set in the year 1990. Three or more of the following: age of onset ≥50, new headache, temporal artery abnormality i.e., thickening, elevated ESR and features of vasculitis on TAB specimens, were associated with a sensitivity of 93.5% and a specificity of 91.2% [24]. However, as these criteria were not diagnostic tests and were limited by the technology available at the time of their development, they need to be supported by imaging such as Colour Doppler Sonography (CDS), MRI, CT, FDG-PET/CT or TAB in order to make a conclusive diagnosis [25]. JG had two of three ACR criteria suggesting they are not sensitive enough to diagnose GCA.

At present, the reported gold standard for diagnosis is the TAB. This involves sampling of the temporal artery through a small incision at the hairline. The predictive value of such a biopsy is affected by vessel length, presence of a contralateral biopsy specimen, prior investigations, and operator experience. Clinical presentation with diplopia, jaw claudication, abnormal temporal artery pulse and anatomy can be the most sensitive indicator of a positive result, alongside an elevated ESR and CRP [26]. However, patients may present without cranial symptoms of GCA. A retrospective multicenter cohort study identified 31 such patients of whom 65% (*n* = 20) were diagnosed via TAB and 10% (*n* = 3) diagnosed using vascular histopathology from other sites. The remaining 25% (*n* = 8) had a negative TAB but a positive FDG-PET/CT [27].

In histological specimens, inflammation can present as skip lesions. Therefore, an adequate length of artery must be sampled to reduce the incidence of false negatives reported to be as high as 42%, which may lead to premature cessation or indeed non-initiation of therapy [28]. A retrospective multi-centre study examining 1821 TAB reports found a TAB length of 5 mm or greater to be associated with increased diagnostic sensitivity [29]. However, another retrospective multi-centre study examining 538 patients found that a cut-off point of 15 mm was associated with increased odds of a positive result by 2.25 (*p* = 0.003), where each millimetre increase in TAB length was associated with an increased odds of a positive result by 3.4% (*p* = 0.024) [30]. Once treatment is initiated, the sensitivity of temporal artery biopsy does reduce gradually over a period of four weeks and can alter the diagnostic yield [31]. This invasive diagnostic modality is therefore associated with a specificity of 100% but low sensitivity. For example, TAB was associated with a sensitivity of 39% in one prospective multi-centre study [15]. A negative TAB in a patient with clinical presentation suggestive of GCA must be interpreted with caution and adjunctive imaging sought.

Less invasive methods of assessment have been proposed. Colour Doppler sonography (CDS) of the temporal artery has been demonstrated in three meta-analyses to have a sensitivity of 68–75% and a specificity of 82–91% [32,33,34]. The presence of arterial wall thickening or a dark hyper-echoic area around the vessel lumen referred to as a periluminal halo representing inflammatory change and oedema of the vessel wall can be diagnostic of temporal arteritis. However, studies suggest that there is a degree of heterogeneity observed in the ascertainment of GCA with CDS possibly in part due to the operator dependent nature of this investigation. In the TABUL prospective multicentre study of 381 new cases of suspected GCA, the sensitivity and specificity of ultrasonography (US) as an alternative to TAB for the of diagnosis GCA was tested. US was found to be more sensitive (54% vs. 39%) and cost-effective for diagnosing GCA but was associated with a lower specificity of 81% [15]. The authors concluded that US cannot replace TAB. However, a combination strategy where all patients underwent an US followed by TAB in scan negative patients, combined with the ascertainment of clinical features as well as the CRP and ESR, increased the likelihood of a firm diagnosis of GCA [15,35].

High resolution Magnetic Resonance Imaging (MRI) of the temporal arteries may be useful for investigating suspected temporal arteritis where there is an absolute contraindication to a TAB. Detection rate is similar to that of CDS [36]. One multi-centre trial (*n* = 185) using T1-weighted images suggested a sensitivity of 78.4% and specificity of 90.4% for the total study cohort, with a sensitivity of 88.7% and specificity of 75.0% for a sub-cohort (*n* = 98) of patients who also underwent a TAB. Diagnostic accuracy was highest in those patients who had received systemic corticosteroids for less than or equal to five days [37].

FDG-PET/CT has also been shown to have a high sensitivity, but low specificity, in establishing a diagnosis in patients with FUO particularly where the diagnosis may be due to non-infectious inflammatory disease or occult infection. This imaging modality is highly beneficial in patients with positive inflammatory markers, reflecting a higher pretest probability of active disease [38]. FDG-PET/CT has been shown to provide high levels of accuracy in diagnosing vasculitis if there is suspected large vessel involvement in either the chest, neck, or abdomen [39]. A meta-analysis (*n* = 57) of patients assessed with FDG-PET/CT showed a sensitivity of 90% and a specificity of 98% for a diagnosis of vasculitis, where vascular tracer uptake greater than that of the liver was taken as representative of disease activity [40]. Furthermore, the sensitivity of FDG-PET/CT in the diagnosis of GCA can remain high up to three days after steroid treatment and decreases after 10 days. This provides clinicians an opportunity to commence corticosteroid treatment promptly without reducing the diagnostic accuracy of FDG-PET/CT [41]. Similarly, the size of the periluminal halo seen on CDS is considerable smaller after 4 days of corticosteroid use [35].

More recently, in acknowledgement of the need for a rapid diagnosis, a consensus opinion of experts from the European League Against Rheumatism (EULAR) taskforce recommend early imaging with CDS and MRI, with FDG-PET/CT as an alternative for patients in whom GCA is suspected. If the diagnosis is not clear after this, additional investigations with TAB are recommended. The task force concluded that all investigations recommended provide high diagnostic value for cranial GCA [42,43].

## 6. Current Management

As vasculitis is an inflammatory process, corticosteroids are the first and often the only treatment offered in the management of GCA. The British Society of Rheumatology (BSR) recommend that in uncomplicated GCA defined as absence of jaw claudication or visual symptoms, oral prednisolone should be initiated at a dose of 40–60 mg once daily.

If evolving signs or symptoms of visual loss during treatment or a history of amaurosis fugax are present, intravenous methylprednisolone (500 mg–1 g) daily for three days can be given, stepped down after this to a maintenance dose of 60 mg oral prednisolone daily alongside ophthalmology input [44,45].

In high risk patients, high dose corticosteroids (60 mg prednisolone) should continue for 3–4 weeks until both inflammatory markers and patient symptoms resolve. The rate of relapse is high especially if corticosteroids are reduced too rapidly in the first few months of treatment. The UK National Institute of Health and Care Excellence (NICE) (cks.nice.org.uk) suggest that when clinical and laboratory features suggest resolution of active disease, the prednisolone dose can reduce by 10 mg every 2 weeks until the patient is taking 20 mg daily, followed by a reduction of 2.5 mg every 2–4 weeks until the patient is taking 10 mg daily, followed by reduction in 1 mg every 1–2 months. After each change the patient should be followed up to exclude any relapse. In the majority of patients the prednisolone can be discontinued after 1–2 years but in patients with chronic relapsing disease, low dose steroids will need to be continued long term [45]. Tapering of steroids, doses, and duration of treatment should be assessed and reviewed on an individual basis. Symptoms should improve within 24–48 h of initiation of corticosteroids; this was seen in our patient. If there is no clinical improvement the diagnosis should be reconsidered.

Both the BSR and NICE advocate treatment with low dose aspirin to prevent cerebrovascular complications; however this is at present supported by expert opinion and level 4 evidence that show a statistically significant reduction in cranial ischaemic complications [46]. It is currently unclear as to the optimum duration of treatment with aspirin, with some advocating an indefinite prescription [47].

Vasculitis is a chronic condition and may require extended courses of treatment. Side effects from glucocorticoid therapy are common and carry with them significant morbidity affecting 60–86% of patients; most commonly dyspepsia, osteoporosis, and diabetes [48]. Gastrointestinal protection should be considered with a proton pump inhibitor. Clinical risk factors and bone density measurement to determine fracture risk will need to be considered in all patients in whom steroids are going to be continued for greater than three months. Vitamin D should be replaced in patients who are deficient. A combination of calcium and vitamin D as well as a bisphosphonate may be commenced in whom the fracture risk is deemed high after considering whether their normal intake of dietary calcium and exposure to sunlight is adequate [45].

Steroid sparing agents are therefore currently being investigated as a method of reducing both total steroid dose and duration of treatment. Methotrexate is currently the most commonly used sparing agent. It has been demonstrated to reduce the risk of relapse and reduce the total cumulative steroid dose [49]. Methotrexate is currently reserved for steroid resistant cases or patients at high risk of corticosteroid side effects. Azathioprine, Cyclophosphamide, Hydroxychloroquine, and TNF-α inhibitors are all ineffective in the long term management of GCA [50].

IL-6 receptor antagonists have been posited as treatment for refractory GCA unresponsive to steroid therapy [25]. Tocilizumab, an IL-6 receptor-alpha inhibitor, has recently been shown to be of benefit in reducing the total length of steroid course. One recent trial (*n* = 30) found that in combination with prednisolone, steroid treatment was stopped >12 weeks prior to those on steroids alone [51]. A further randomised controlled trial by Stone et al. reported that Tocilizumab in either weekly or alternate-weekly administrations alongside a 26 week prednisolone tapered course versus placebo (prednisolone only taper of 26 or 52 weeks) was associated with a significant (*p* < 0.001) increase in remission rates at 52 weeks of up to 42%. Tocilizumab patients also had a reduced cumulative steroid dose of between 44–52% when compared to placebo [52]. Both Ustekinumab, an IL-2/IL-23 inhibitor and Anakinra an IL-1 blocker, have steroid sparing properties but require further investigation in larger clinical trials.

## 7. Discussion

The vasculitides are great mimics, particularly in older people in whom comorbidities and atypical presentations often throw up red herrings. A FUO/sepsis was high on the differential diagnosis in JG. A FUO can be caused by many infections as well as non-infectious inflammatory diseases which include autoimmune diseases, vasculitides, and malignancy; including paraneoplastic syndromes [53]. In the case of JG, age, un-resolving constitutional malaise and in retrospect, a cough and sore throat were the only supporting features in his presentation that could have pointed towards a vasculitis [54]. He did not have typical features of GCA: headache and scalp tenderness, monocular or binocular visual symptoms, jaw claudication, or a raised ESR [5]. He had no symptoms suggestive of pulmonary infiltration or glomerulonephritis. There was no history nor clinical features of PMR, mononeuritis multiplex, or unexplained ischaemic events. However, in retrospect, a normochromic normocytic anaemia developing 15 days after admission (Hb 115 g/L), thrombocytosis and elevated alkaline phosphatase were present in our patient (Table 1). Literature suggests that up to 1/3 of patients with GCA have deranged liver function tests.

Eosinophilia was a striking feature in JG and influenced extended serological testing. Although there is a link between hyper-eosinophilic syndrome and juvenile temporal arteritis [55], there are no strong clinical associations between peripheral blood eosinophilia and adult GCA [56]. The eosinophilia in our case may have simply reflected the general elevation in inflammatory cell milieu.

A delay in diagnosis may have lasting life changing effects in terms of neuro-ophthalmologic and cardiovascular morbidity. Equally important is an accurate diagnosis as commencement of high dose corticosteroids based on symptoms mean patients may be exposed to prolonged high doses that risk unwanted effects such as weight gain, diabetes, altered body habitus, osteoporosis, cataracts, thin skin, altered mood, and hypertension [57]. In this vein, long term management aims are to prevent complications associated with the disease process of GCA or complications related to steroid use [45]. Treatment is often given for approximately two years and whilst steroid use decreases, 30–50% of patients may have spontaneous exacerbations in the first two years after diagnosis. Thus, long term management with immunosuppressive medication needs to be considered as studies have shown that there is an increased incidence of large vessel complications such as aortic aneurysm/dissection, large-artery stenosis, as well as valvular heart disease i.e., aortic regurgitation [58]. Complications can be categorized into: 1. Disease related e.g., visual loss, stroke, and cardiovascular disease. 2. Late complications e.g., aortic dissection and development of aortic aneurysm. 3. Complications associated with long term steroid use [58]. JG developed a below knee DVT approximately 3 months after the diagnosis of GCA, whilst still on treatment and follow up. This is a known, potentially serious complication of GCA. In one observational cohort study (*n* = 909), the highest incident rate ratio for the development of DVT was within the first year of diagnosis, reducing in subsequent years. This was found to be independent of other risk factors, age, or gender [59] and may lend support to the use of routine prophylaxis against venous thromboembolism and ischaemic events post diagnosis. Further randomised trials are required to fully clarify the potential risks and benefits of such therapy.

It is therefore recommended that patients with GCA have follow up every three to six months but more frequently if they are being treated for symptoms suggesting relapse or adverse events of the disease or treatment. A full hematological, biochemical, and inflammatory screen are essential prior to medical follow up as well as two yearly ascertainment of large vessel pathology through chest radiography or echocardiography [13]. In addition, repeat FDG-PET/CT may have some value in the ascertainment of ongoing vascular inflammation in long term follow up [60,61].

## 8. Conclusions

This case highlighted the diagnostic dilemma driven by a host of non-specific symptoms. It is clear that a right, timely, and structured fast track diagnostic pathway is needed for patients in whom GCA is suspected. As frustrating as it was for the patient and the multidisciplinary team, a major strength in JG’s care was that all potential diagnoses were systematically excluded whilst being aware of any potential red flag symptoms. The case was a learning experience for the medical teams in terms of perseverance, MDT working, and the importance in starting treatment early. We are pleased JG was discharged on the correct treatment and robust follow up is in place. Non-specific symptoms, ‘sepsis’ or FUO are commonly seen in older people presenting acutely given the increased prevalence of comorbid conditions and frailty. We take from this case to always consider vasculitis in cases of diagnostic uncertainty in older people with constitutional symptoms, raised inflammatory markers, and inconclusive initial imaging.

## 9. Patient’s Perspective

We sought JG’s view of his admission and the paragraph below is taken from his reply.

“*The near daily explanations of my various symptoms by you and your team always proved to be a little mysterious and I have therefore been pleased and grateful to trust your knowledge and integrity and not to worry myself unduly regarding my lack of a physician’s skill. For that reason I am indebted to the entire team for devoting so much of their time and energy into identifying my health problem and to its continuing cure…my stay in hospital remains a blur to me but I shall always be thankful to all who did so much to make me comfortable*”.

## Figures and Tables

**Table 1:** Serum values.

	Units of Measurement	Normal Range	Patient Values on Admission	Patient Values on Discharge
Haemoglobin	g/L	130–170	138	121
White cell count	10^9^/L	4.0–11.0	26.2	11.2
Platelets	10^9^/L	150–400	576	308
Neutrophil	10^9^/L	2.0–7.5	21.9	7.1
Eosinophil	10^9^/L	0.0–0.5	1.7	0.7
ESR	mm/h	1–30	22	5
CRP	mg/L	0–7.5	245	33
Urea	mmol/L	2.5–7.8	24.2	12.4
Creatinine	μmol/L	80–115	154	130
Creatine Kinase (CK)	U/L	40–320	83	N/A
ALT	U/L	10–40	30	11
ALP	U/L	30–130	185	88
Albumin	g/L	35–50	19	25

ESR: Erythrocyte sedimentation rate; CRP: c-reactive protein; ALT: alanine transaminase; ALP: alkaline phosphatase.

## Abbreviations

GCA	Giant cell arteritis
TAB	Temporal artery biopsy
MDT	Multidisciplinary team
ESR	Erythrocyte sedimentation rate
VZV	Varicella zoster
CRP	c-reactive protein
HLA-DRB	Human leucocyte antigen, nucleotide sequence DRB
FDG-PET/CT	fluorodeoxyglucose positron emission tomography
IL-	Interleukin
FUO	Fever of unknown origin
Th-	T-helper cell
ALP	Alkaline phosphatase
IFN-γ	Interferon gamma
CK	Creatine Kinase
MRI	Magnetic resonance imaging
CT	Computerised tomography
BSR	British Society for Rheumatology
ANCA	Anti-neutrophil cytoplasm antibodies
NICE	National Institutes for Health and Care Excellence
TNF	Tumour necrosis factor
DVT	Deep vein thrombosis
TABUL	The Role of Ultrasound Compared to Biopsy of Temporal Arteries in the Diagnosis and Treatment of Giant Cell Arteritis: a diagnostic accuracy and cost-effectiveness study
